# Nanoemulsion as a promising drug delivery strategy for effective eradication of *Helicobacter pylori*: current insights

**DOI:** 10.1007/s13346-025-01986-7

**Published:** 2025-10-13

**Authors:** Moumita Saha, Ashutosh Gupta, Shivani Kunkalienkar, Namdev Dhas, Shiran Shetty, Abhishek Gupta, Srinivas Mutalik, Nandakumar Krishnadas, Raghu Chandrashekar, Nagalakshmi Narasimhaswamy, Sudheer Moorkoth

**Affiliations:** 1 Department of Pharmaceutical Quality Assurance, Manipal College of Pharmaceutical Sciences, Manipal Academy of Higher Education, Manipal, 576104 Karnataka India; 2 Department of Pharmaceutics, Manipal College of Pharmaceutical Sciences, Manipal Academy of Higher Education, Manipal, 576104 India; 3 Department of Gastroenterology and Hepatology, Kasturba Medical College, Manipal, Manipal Academy of Higher Education, Manipal, 576104 India; 4 School of Pharmacy, Faculty of Science and Engineering, University of Wolverhampton, Wulfruna Street, WV1 1LY UK; 5 Department of Pharmacology, Manipal College of Pharmaceutical Sciences, Manipal Academy of Higher Education, Manipal, 576104 India; 6 Department of Pharmaceutical Biotechnology, Manipal College of Pharmaceutical Sciences, Manipal Academy of Higher Education, Manipal, 576104 India; 7 Department of Microbiology, Faculty of Medicine, Manipal University College Malaysia (MUCM), Bukit Baru, Melaka, 75150 Malaysia

**Keywords:** Antibiotic resistance, Bioavailability, Eradication, Helicobacter pylori, Nanoemulsion

## Abstract

*Helicobacter pylori* (*H. pylori*) have infected about 50% of the world’s population and is a leading cause of gastrointestinal diseases, including gastritis, peptic ulcer, and stomach cancer. Current treatment regimens often fail to completely eradicate the bacteria due to the failure of antibiotics to penetrate into stomach’s inner mucosa, where the bacteria reside. Additional factors such as the ability of the organism to neutralize the stomach’s acidic environment and biofilm formation further contribute to treatment failure leading to antibiotic resistance. These challenges underscore the urgent need for new treatment options and strategies to combat *H. pylori* effectively. The current review delivers an overview of the pathophysiology of *H. pylori*, the limitations of the current regimens, and the potential of nanoemulsion as a smart carrier addressing the limitations associated with *H. pylori* treatment. The nanoemulsion offers specific advantages like mucoadhesion potential, targeted delivery, controlled release, and co-delivery options that ultimately results in an enhancement of bioavailability of the antibiotics to *H. pylori*, which resides in the inner walls of the stomach mucosa. Further, the ability of nanoemulsions to encapsulate the drug molecules helps in protecting the antibiotics from the stomach acidity facilitating drug stability. In conclusion, the review highlights the importance of tapping this unexplored potential of nanoemulsion as a promising drug delivery option for the treatment of *H. pylori* infection.

**Graphical abstract:**

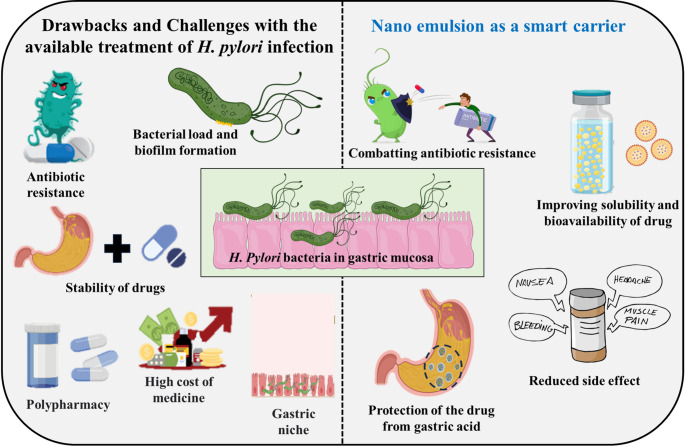

## Introduction

**Fig. 1:**
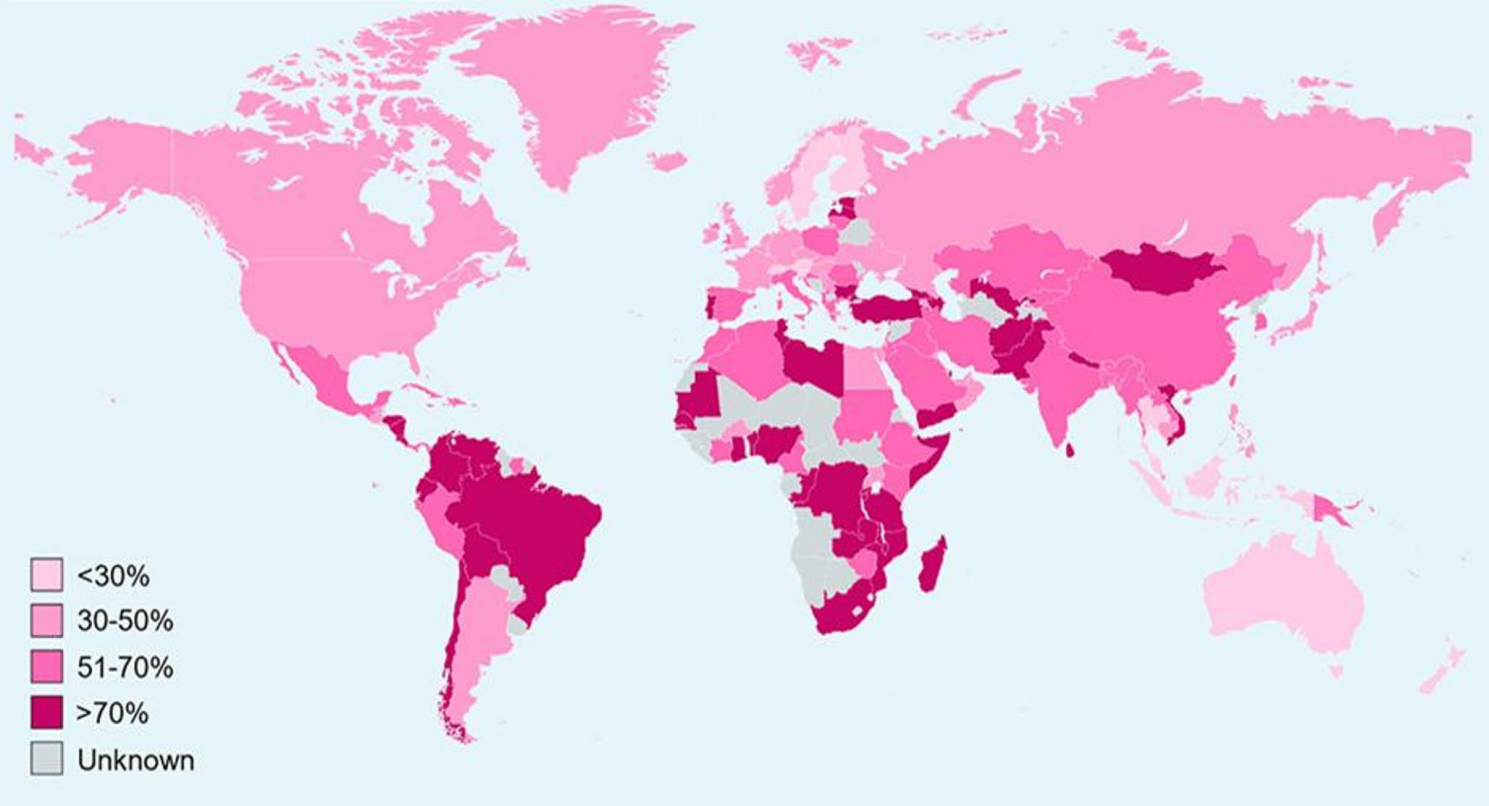
Global prevalence of *H. pylori *infection [Adapted and reproduced with permission from [24]]

*Helicobacter pylori (H. pylori*), is a Gram-negative, microaerophilic, S-shaped bacterium that infects the gastric lining and upper part of the small intestine [1–3]. *H. pylori* is reported to infect more than 50% of the world’s population with more prevalence in developing countries. *H. pylori* is known to be a significant contributing factor to non-atrophic and atrophic gastritis leading to the development of peptic ulcers [4, 5]. Untreated gastric ulcers can lead to gastric mucosa-associated lymphoid tissue lymphoma and gastric adenocarcinoma [6, 7]. Studies have shown that *H. pylori* infections are responsible for around 89% of all gastric malignancy [8–10]. Other disease conditions associated with *H. pylori* infection include hepatobiliary diseases, pain and discomfort in the upper abdomen, gastroesophageal reflux disease, iron deficiency anaemia, idiopathic thrombocytopenic purpura, vitamin B_12_ deficiency, myocardial infarction, anti-phospholipid syndrome, insulin resistance, diabetes mellitus and liver disease [11–15]. *H. pylori* is primarily spread through oral-oral or fecal-oral routes [16–18]. While many individuals with *H. pylori* infection may remain asymptomatic, they can play a vital role in transmission of the bacteria. Contaminated water and food products especially dairy products, plays a major role in transmission of this bacteria [16, 19, 20]. Individual differences in infection severity are caused by bacterial variables, host variables, and environmental variables. Environmental factors like poor hygiene, sanitation, and socioeconomic condition can contribute in higher prevalence rates of *H. pylori* infection [21]. Dietary habits, genetic susceptibility, and age are other host associated factors that affect the infection [22]. There are significant differences in the occurrence of *H. pylori* infection by age, race, ethnicity, socioeconomic position, and geographic location [19]. A representation of global prevalence is shown in Fig. 1. The global market for *H. pylori* infection treatments is around USD 638.3 million and is expected to increase to USD 1,260 million between 2023 and 2033 at a compound annual growth rate of 7.04%. Recently, Europe is being regarded as the second largest market for *H. pylori* infection treatment with a 30% revenue rate [23].

Despite the existing treatment options, management of *H. pylori* infections is a major clinical challenge. The reasons for treatment failure attributed to the peculiar pathophysiology of the bacteria leading to incomplete eradication from the system [25]. The bacteria are well adapted to survive in the harsh acidic pH of the stomach, in the aerobic and anerobic conditions of the extracellular space of the mucosa and in the intracellular vacuoles. This ability of the organism prevents the antibiotics from eradicating the bacterium completely thus developing resistance [4, 26]. Due to this reason, clinicians often rely on polypharmacy for treatment, which can be inconvenient for patients, contributing to antibiotic resistance. This alarming rise in antibiotic resistance underscores the need for innovative drug delivery solution for the bacterial infection management. Leveraging the proven use of nanoemulsion (NE) in food and cosmetics, NE are gaining traction as a novel drug delivery system for *H. pylori* infections. This review consolidates current knowledge on *H. pylori* and highlights NE’s potential to enhance antibiotic therapy through targeted delivery and improved drug penetration.

## H. pylori pathophysiology

**Fig. 2:**
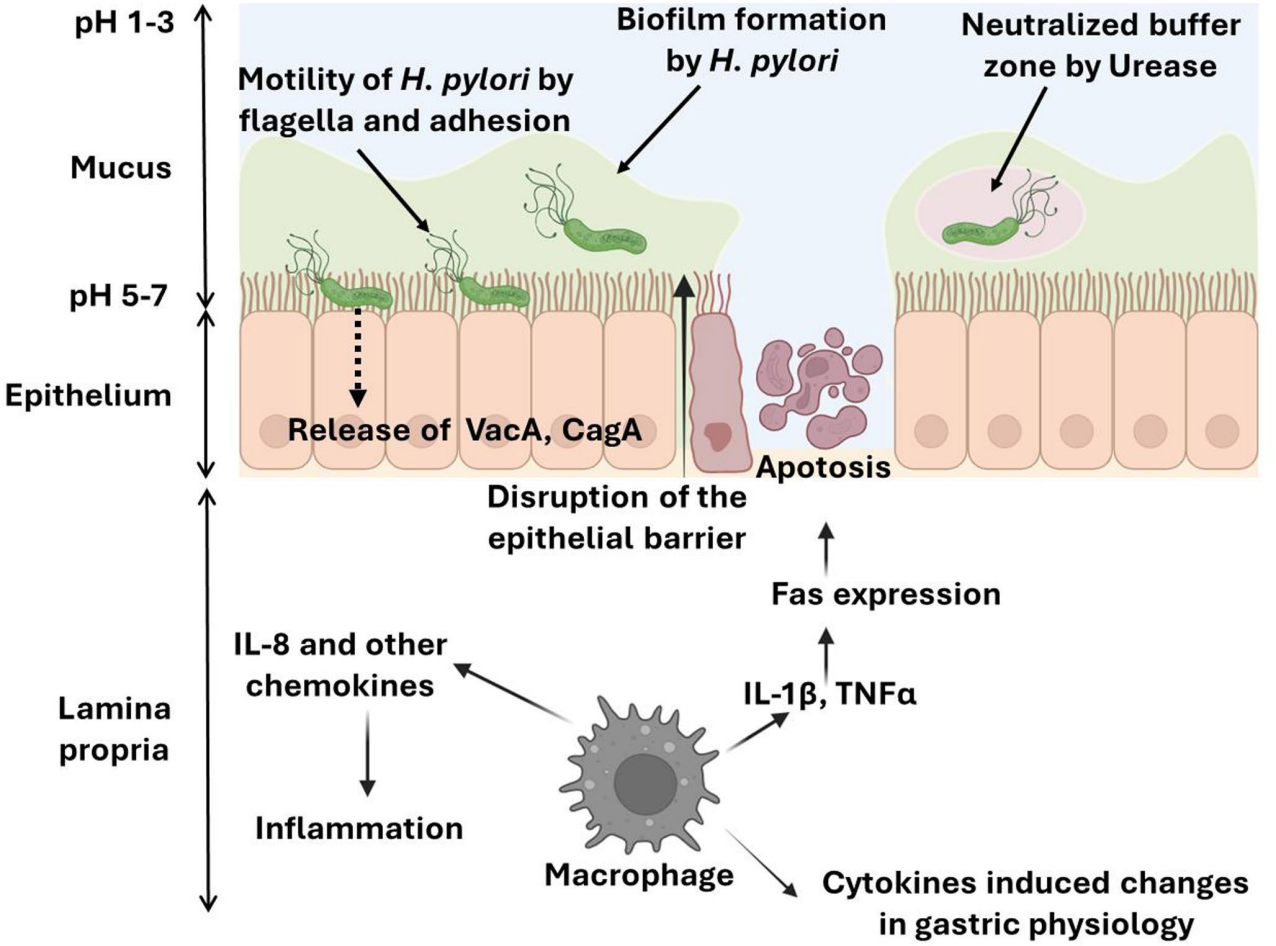
A pictorial representation of *H. pylori* pathogenesis and disease induction

Colonization: After getting into the human body, *H. pylori* survive the gastric acidic environment with the help of urease enzyme. It hydrolyses urea to produce ammonia, which neutralizes stomach acid in the vicinity of *H. pylori*, creating a less acidic microenvironment that promotes bacterial viability and colonization. Urease also plays a role in simulating bacterial nutrition in the stomach [27, 28]. After surviving the stomach’s acidic environment, the bacteria rapidly swim through the protective mucus layer that covers the gastric epithelial cells with the help of the flagella and resides inside at the inner layer of the mucus membrane [28–30]. *H. pylori* attach itself to particular stomach epithelial cell receptors with the help of adhesins, such as sialic acid binding adhesin, blood group antigen binding adhesin, and adherence-associated lipoprotein A and B. This adherence to gastric epithelial cells facilitates colonization and infection of *H. pylori* [30, 31]. Protein toxin produced by the organism called vacuolating cytotoxin A (VacA) causes vacuoles to develop in host cells, disrupts cellular functions, and modulates the host immune response. VacA-mediated cytotoxicity contributes to tissue damage and inflammation, which leads to the development of peptic ulcer disease (PUD) and gastric adenocarcinoma [32]. Different pathways of *H. pylori* infection pathogenicity and disease induction are illustrated in Fig. 2.

Immune escape: *H. pylori* has shown various mechanisms for evading the host’s innate immune response [33]. The flagellins and lipopolysaccharides of *H. pylori* helps to avoid recognition by human pattern recognition receptors like Toll-like Receptor 5 and Toll-like Receptor 4 and contribute to immune evasion facilitating its persistent colonization [34]. This alters the cellular homeostasis resulting in the release of cytokines and chemokines. Nuclear factor-κB (NF-κB) activation, for instance, increases the expression and release of interleukins like IL-8 and other chemokines [33, 35]. *H. pylori* also induce the activation of T cells (including TH17 (T helper 17), TH1 (T helper 1), and Treg (regulatory T) cells) in the stomach mucosa. An investigative study on vaccination in animal models implied that both TH1 and TH17 cells play a role in modifying the immunity resulting in *H. pylori* infection [2, 36].

## Current treatment regimens

Current treatment strategy uses a combination of antibiotics and an acid-suppressing agent [37]. The choice of antibiotics is influenced by factors such as regional bacterial resistance patterns and patient history of antibiotic exposure [38]. The first line treatment regimen typically includes amoxicillin, clarithromycin, and a proton pump inhibitor (PPI) for a duration of 14 days. However, the efficacy of this regimen can be impacted by clarithromycin resistance. Nowadays, quadruple therapy consisting of PPI, amoxicillin, clarithromycin, and a nitroimidazole has been found most effective for overcoming the antibiotic resistance [2].

In case of high antibiotic resistance, second-line therapy is used, which involves bismuth, tetracycline, metronidazole or tinidazole and PPI for 7–14 days [39]. An outstanding rate of *H. pylori* eradication was demonstrated by the bismuth quadruple therapy (more than 80%) and is considered a good option in patients with macrolide exposure or penicillin allergies. However, the unavailability of bismuth, rising resistance to metronidazole, and adverse effects like black stool have limited the use of bismuth quadruple therapy [40]. Alternative regimens involve levofloxacin-based treatment for 10 to 14 days and are considered a substitute for the clarithromycin triple therapy. This treatment regimen contains PPI, amoxicillin, and levofloxacin [25, 41]. Table 1 compares commonly recommended first-line regimens and widely used second-line strategies for the eradication of *H. pylori* infection. The treatment outcomes, drug choices, and rationale for regimen selection are supported by recent reviews and clinical trial data, emphasizing the importance of local antibiotic resistance patterns when considering first- and second-line management approaches for *H. pylori* infection.

When the above mentioned treatments do not work or when the other treatment causes antibiotic resistance, the patients are prescribed to administer third-line treatment [25]. This third line treatment is rifabutin-based therapy consisting of PPI, rifabutin, and amoxicillin administered twice a day for 10–14 days [42]. Rifabutin is a derivative of rifampicin, which can cause serious myelotoxicity and should be used only as rescue therapy. Rifaximin is also used as a third-line treatment and has shown an eradiation efficiency of 50–60% without combining with amoxicillin, clarithromycin, or levofloxacin.

**Table 1:** Comparison of first line and second line treatment strategies for *H. pylori* infection management

Aspect	First-line treatment	Second line treatment
**Goal**	Initial eradication of *H. pylori*.	Eradication after failure of first-line therapy.
**Common regimens**	• **Bismuth Quadruple Therapy (BQT)**: PPI + Bismuth + Tetracycline + Metronidazole/Tinidazole (10–14 days) • **Non-Bismuth Quadruple (Concomitant) Therapy**: PPI + Clarithromycin + Amoxicillin + Metronidazole/Tinidazole (10–14 days) • **Sequential Therapy**: PPI + Amoxicillin (first 5–7 days) then PPI + Clarithromycin + Metronidazole/Tinidazole (next 5–7 days) • **High-Dose Dual Therapy (HDDT)**: High-dose PPI + Amoxicillin	• **Bismuth Quadruple Therapy (BQT)**: (if not used first-line) • **Levofloxacin-based Triple Therapy**: PPI + Levofloxacin + Amoxicillin (10–14 days) • **Concomitant Therapy**: (if not used first-line) • **Rifabutin Triple Therapy**: PPI + Amoxicillin + Rifabutin
**Selection factors**	• Local clarithromycin resistance rates (≥ 15–20% often precludes clarithromycin triple therapy) • Patient allergies and tolerability • Treatment availability	• Antibiotics used in a failed first-line regimen • Local resistance patterns (especially to levofloxacin) • Patient allergies
**Resistance impact**	• Standard clarithromycin triple therapy less effective with high clarithromycin resistance. • BQT is generally unaffected by clarithromycin resistance.	• Effectiveness is significantly impacted by resistance to previously used or new antibiotics (e.g., levofloxacin resistance).
**Guideline treatment approach**	Increasingly emphasises on susceptibility-guided (tailored) therapy based on resistance testing (PCR/culture for clarithromycin, metronidazole, levofloxacin).	Aims to use different antibiotic classes than those in the failed first-line regimen. Often relies on previous antibiotic history.
**Efficacy**	Higher eradication rates compared to second-line regimens.	Generally lower eradication rates than first-line regimens.
**Success rate**	Aim for ≥ 90% eradication in most regions (lower where resistance is high)	Varies; several regimens exceed 85–90% efficacy if chosen based on resistance and prior regimen
**Advantage**	• Well-studied, standardized regimens • Good tolerability (in most cases)	• Tailored to patient’s previous therapy and resistance • Multiple effective options (drug classes differ from first-line)
**Limitations**	• Increasing antibiotic resistance (especially clarithromycin). • Need for resistance testing infrastructure. • Declining efficacy due to rising resistance (especially clarithromycin and metronidazole)	• Multidrug resistance. • Limited effective options after multiple failures. • Increased pill burden and potential side effects (especially bismuth quadruple regimens) • Some regimens may not be suitable with persistent fluoroquinolone or tetracycline resistance
Note	Perform susceptibility-guided therapy when feasible, especially in high-resistance areas	Antimicrobial susceptibility testing can improve outcomes when available

### Drawbacks associated with the current treatment regimes

Antibiotic resistance is a main concern for the failure of existing therapy regimens [25, 38, 43]. For clarithromycin, amoxicillin, metronidazole, tetracycline, and levofloxacin, the primary resistance rates are approximately 34–55%, 15%, 69–71%, 18% and 18–28%, respectively [25]. The reason for antibiotic resistance is due to the limited reach of antibiotics and the duration of antibiotics exposure at the site where the *H. pylori* reside. Most antibiotics are active against this multiplying organism. The slow growth of *H. pylori* organisms can also have an advantage in their survival [44]. Current treatment regimens fail to eradicate bacteria, and this incomplete eradication leads to persistent infection, recurrence of symptoms, and resistant strains of *H. pylori* [45, 46]. Table 2 summarizes the mechanisms of antibiotic resistance in *H. pylori*.

Other drawbacks include the gastric niche, problems due to side effects, reinfection risk, presence of ulcer in the stomach, impact of long-term treatment on the gut microbiota, and the complexity of regimens. Some drugs degrade in gastric environment because of enzymatic action and hydrolysis of drugs. Additionally, drugs may ionize in the stomach’s acidic environment, which would stop them from cell permeation [25]. Side effect due to antibiotics and other medications used in *H. pylori* treatment is another challenge. It is a known fact that antibiotic-based treatments not only target *H. pylori* but also affect the balance of the gut microbiota. By affecting the gut flora, long-term treatment affects the digestive system and leads to metabolic abnormalities, including gut microbiota dysbiosis [25]. Disruption of the gut microbiome can have long-term consequences on digestive health and immune function [47]. Complexity of regimens and accessibility of medications are the other drawbacks. Current treatment regimens for *H. pylori* often involve multiple medications taken concurrently for a specified duration which can be costly. This polypharmacy can lead to medication non-adherence, especially in cases where patients find it challenging to follow the prescribed regimen [24]. The cost factor, along with issues of accessibility to healthcare services and medications, can pose challenges in effective *H. pylori* management [48].

**Table 2:** Antibiotic resistance mechanisms in *H. pylori* bacterium

Antibiotics	Mechanism of action against *H. pylori*	Mode of Resistance	In-vivo/ Biological Change	References
**β-Lactams** (e.g. amoxicillin)	Disrupts the bacterial cell wall synthesis	Enzymatic degradation	Bacteria produce enzymes called beta-lactamases, which breaks down the beta-lactam ring of drugs, neutralizing its antibacterial activity.	[49]
		Target modification	Mutations in PBPs^(I)^ (i.e. PBP1A, PBP2, PBP3) decrease drug binding, maintaining cell wall synthesis even in the presence of the antibiotic.	[38, 50]
		Drug efflux	Efflux pumps reduce intracellular concentration of drugs, preventing it from reaching its target in effective concentrations.	[51]
		Reduced permeability	Gram-negative bacteria alter their outer membrane porin channels (HopC^(II)^ and HofH^(III)^), reducing uptake of antibiotics like amoxicillin into the cell.	[52]
		Plasmid acquisition	Many resistant genes are carried on plasmids, which can be transferred between bacteria through horizontal gene transfer, spreading resistance rapidly.	[53]
**Macrolides** (e.g. clarithromycin)	Binding to the 50 S ribosomal subunit of the bacteria, effectively inhibiting protein synthesis by preventing the peptidyl transferase enzyme from adding new amino acids to the growing peptide chain, ultimately halting bacterial growth	Drug target-mediated resistance	Mutations in the 23 S rRNA^(IV)^ or ribosomal proteins (such as L4 and L22) can reduce the binding affinity of macrolides to the ribosome.	[54]
		Methylation	The ERM^(V)^ genes encode methyltransferases that cause methylation of adenine residues in the 23 S rRNA, which blocks macrolide binding and confers resistance.	[55, 56]
		Enzymatic modification	Bacteria produce macrolide-hydrolysing enzymes that can modify or degrade macrolides, neutralizing their antibacterial activity.	[57]
**Nitroimidazoles** (e.g. metronidazole, tinidazole)	Acts as prodrugs that are activated within the bacterial cell through a reduction process by an enzyme called nitro-reductase, generating toxic free radicals that damage bacterial DNA, leading to cell death	Reduced drug activation	Nitroimidazoles require activation through reduction by bacterial enzymes. Mutations or downregulation of these enzymes, such as nitro-reductases, can prevent the activation of the drug, rendering it ineffective.	[58]
		DNA repair mechanisms	Enhanced DNA repair mechanisms can allow bacteria to survive the DNA-damaging effects of nitroimidazoles. Upregulation of genes involved in DNA repair can contribute to increasing resistance rate.	[59]
		Biofilm protection	Enhanced DNA repair mechanisms can allow bacteria to survive the DNA-damaging effects of nitroimidazoles. Upregulation of genes involved in DNA repair can contribute to resistance.	[60]
**Tetracycline** (e.g. tetracycline, doxycycline)	Binds reversibly to a pocket in the 30 S subunit of bacterial ribosomes containing 16 S rRNA, causing bacteriostatic and bactericidal effects by inhibiting protein synthesis and bacterial growth	Ribosomal Protection	Bacteria produce proteins, encoded by genes such as tet(M)^(VI)^ and tet(O)^(VII)^, that protects the ribosome from tetracycline binding and maintaining protein synthesis.	[61]
		Efflux Pump overexpression	The expression of efflux pumps encoded by the tet(A), tet(B)^(VIII)^, and tet(K)^(IX)^ genes actively transport tetracycline out of the bacterial cell, reducing its intracellular concentration.	[62]
		Target Modification	Mutations in the genes encoding the 16 S rRNA of the 30 S ribosomal subunit can reduce the binding affinity of tetracycline allowing the bacteria to continue synthesizing proteins despite the presence of the antibiotic.	[63]
**Fluoroquinolones** (e.g. levofloxacin)	Inhibits the bacteria’s DNA gyrase enzyme, which is crucial for DNA replication, effectively stopping the bacteria from dividing by interfering with its DNA structure through binding to the DNA-gyrase complex	Target modification	Genetic mutations in GyrA^(X)^, GyrB^(XI)^, parC^(XII)^, and parE^(XII)^ genes lead to structural changes in DNA gyrase and topoisomerase IV, preventing effective binding of fluoroquinolones.	[64]
		Efflux and Reduced Uptake	Efflux pumps of RND^(XIII)^ family, actively transport fluoroquinolones out of the bacterial cell, while modifications in porin channels decreases drug entry.	[65]
		Plasmid-mediated resistance	Plasmids carry genes that encode proteins capable of protecting DNA gyrase and topoisomerase IV from fluoroquinolones or produce enzymes that modify the antibiotic.	[66]
		Drug target-mediated resistance	Mutations in the rpoB^(XIV)^ gene, which encodes the beta subunit of RNA polymerase which alter the binding site of rifamycin, reducing its affinity for the enzyme and allowing RNA synthesis to continue.	[67]
**Rifamycin** (e.g. rifabutin)	Inhibits the bacteria’s DNA-dependent RNA polymerase, essentially blocking the process of bacterial RNA synthesis and preventing the bacteria from replicating	Efflux and reduced uptake	Efflux pumps encoded by certain plasmid-borne genes, actively transport rifamycin out of the bacterial cell, while changes in membrane permeability can limit drug entry.	[68]
		Genetic Mutations	Mutations in genes on plasmids, transposons, or integrons that encodes nitro-reductases or other relevant enzymes reduce the activation and efficacy of nitrofurans.	[69, 70]
**Nitrofurans** (e.g. furazolidone)	Enters the bacterial cell and converts by bacterial enzymes (nitro-reductases) into toxic reactive metabolites which disrupt multiple cellular processes including DNA, RNA, and protein synthesis, ultimately leading to bacterial cell death	Metabolic Adaptations	Bacteria can alter metabolic pathways to reduce the activation of nitrofurans, allowing bacterial survival.	[71]
		Efflux Pump Overexpression	Efflux pumps, such as MFS^(XV)^ and RND family, transport nitrofurans out of the bacterial cell.	[72]

**Note: -** (I) PBP - penicillin-binding proteins, (II) HopC - *Helicobacter pylori* outer membrane protein C, (III) HofH - *Helicobacter* outer membrane protein family, (IV) rRNA - ribosomal RNA, (V) ERM- erythromycin ribosome methylation, (VI) tet (M) - tetracycline resistance gene, (VII) tet (O) - Tetracycline resistance protein, (VIII) tet (B) - tetracycline efflux protein, (IX) tet (K) - tetracycline efflux protein, (X) GyrA - DNA gyrase subunit A, (XI) GyrB - DNA gyrase subunit B, (XII) par – Protease activated receptors, (XIII) RND - Resistance-Nodulation-Division, (XIV) rpoB – resistance determining region, (XV) MFS - major facilitator superfamily

## Use of nanomedicines in H. pylori treatment

Nanomedicine offers a diverse array of nanoscale drug delivery systems, including systems such as NE, polymeric nanoparticle, liposomes, metallic nanoparticle, and targeted or stimuli-responsive systems etc. Each approach has distinct features regarding drug loading, targeting, protection of the drug, and controlled release profiles. For instance, liposomes and polymeric nanoparticles can concentrate antibiotics directly at the infection site, enabling sustained release and active targeting, which reduces antibiotic resistance and enhances bacterial eradication compared to conventional therapies [73, 74]. Metallic nanoparticles show direct antimicrobial effects, such as generating reactive oxygen species (ROS), while lipid nanoparticles can inhibit biofilms via targeted drug delivery [75, 76]. Surface modification strategies further improve nanoparticle bioavailability and enable synergistic interactions with antibiotics. Innovations like membrane-coated nanoparticles achieve precise, localized, and sustained drug release, significantly increasing drug exposure to bacteria [77, 78]. Targeted nanoparticles like chitosan or heparin-conjugated nanoparticles, tailored to deliver antibiotics to the gastric niche, increasing mucosal adhesion and site-specific release, showing reduced toxicity, greater biofilm inhibition, lower resistance induction, and better eradication rates in preclinical studies [79–82]. In general, all nanoparticle-based systems enhance drug efficacy by overcoming key challenges— poor drug solubility, gastric instability, limited targeting, and rising resistance—by facilitating deeper cellular penetration, triggered release, biofilm disruption, and ROS generation. They localize antibiotics at the infection site, minimize acid/enzyme degradation, and can be tailored for mucoadhesion, pH-responsiveness, or surface targeting [80, 83, 84]. Various nanocarrier systems have been explored for the treatment of *H. pylori* infections. Table 3 presents the information on the nanocarriers for drug delivery in *H. pylori* treatment that have been reported as on date. Among these approaches, NE stand out for their relatively simple formulation, adaptability to both hydrophobic and hydrophilic drugs, and suitability for oral delivery, making it a practical and versatile candidate for *H. pylori* infection treatment.

**Table 3:** Nanocarriers for drug delivery in *H. pylori* treatment

Nanocarrier Type	Drug encapsulated	Key Features/Mechanism	References
**Chitosan nanoparticles**	Amoxicillin, clarithromycin, berberine	Mucoadhesive, controlled/pH-responsive release, biofilm disruption	[85–87]
**Polymeric nanoparticles (PLGA**^(I)^, **PAA**^(II)^, **etc.)**	Amoxicillin, clarithromycin, metronidazole	Biodegradable, sustained release, mucus penetration	[85–88]
**Liposomes**	Amoxicillin, furazolidone, linolenic acid	Biocompatible, can encapsulate hydrophobic/hydrophilic drugs, mucus penetration	[85, 89]
**Solid lipid nanoparticles (SLN)**,** nanostructured lipid carriers (NLC)**	Clarithromycin, DHA^(III)^, antibiotic-free SLN	Enhanced drug stability, innate antibacterial action; disrupt bacterial membrane	[90]
**Nanoemulsions (NE)**	Essential oils, plant extracts, antibiotics	Improved penetration, increased solubility/bioavailability	[87, 88, 91]
**Urea-coated nanoparticles**	Amoxicillin	Utilizes *H. pylori* UreI channel for targeted delivery	[92]
**Glycan/lectin-conjugated nanoparticles**	Antibiotics, lectin-peptide conjugates	Block adhesion to gastric mucosa/cell surface, targeted binding	[92, 93]
**Metal-based nanoparticles**	Bismuth, silver, zinc oxide, gold	Metal ion release, ROS production, direct bactericidal and anti-biofilm	[76, 87, 88, 94]
**Rhamnolipid chitosan hybrid nanoparticles**	Amoxicillin, clarithromycin	Biofilm penetration and destruction, mucoadhesion and mucus permeation	[95]
**Superparamagnetic iron oxide nanoparticles (SPIONs)**	Amoxicillin, chitosan/polyacrylic acid	Magnetic targeting, enhanced retention, improved membrane penetration	[86]
**Nanocomposites (e.g.**,** clay**,** montmorillonite**,** MOF**^(IV)^	Metronidazole, copper MOF	Mucus-penetration, membrane disruption, multi-functional action	[96, 97]
**Photothermal/sonodynamic nanoparticles**	Photosensitizers, antibiotics	Localized ROS generation, enhanced bactericidal effect via external stimuli	[85, 97]
**Dendrimers**	Antibiotics, gene therapy agents	Highly branched, high loading, multivalency for targeting	[85]
**Lipid-polymer hybrid nanoparticles (LPNs)**	Clarithromycin, mixed drugs	Mucus and biofilm penetration, enhanced payload delivery	[95]

**Note: -** (I) PLGA - poly (lactic-co-glycolic acid), (II) PAA - polyacrylic acid, (III) DHA - docosahexaenoic acid, (IV) MOF - metallic organic frameworks

## Nanoemulsion for the effective eradication of H. Pylori

NE is a biphasic, isotropic, homogeneous, and kinetically stable dispersion system of two immiscible materials, usually liquids, with a droplet size in the nanoscale range (20–200 nm) [98, 99]. It is a type of emulsion that contains small droplets of one liquid scattered throughout another, stabilized by a mixture of surfactants that reduces the surface tension between the two liquids [99]. NE exhibit distinct advantages due to their unique characteristics like kinetic stability, low polydispersity, compact size, optical transparency, and tunable rheology [99–101]. More importantly, NE can be prepared using relatively simple and scalable manufacturing techniques, such as high-pressure homogenization, sonication, or microfluidization, rendering them suitable for large-scale production across industries [98]. The nanometre-sized droplets and large surface area facilitate improved solubility and dispersion of poorly water-soluble or hydrophobic compounds (for e.g. BCS class II and class IV drugs) within the aqueous continuous phase. Their lipophilic interior makes them a better option for transporting lipophilic compounds than liposomes [102, 103]. NE also permits for the targeted delivery by modifying the surface properties or composition of the NE droplets [104, 105]. To allow for real-time medication delivery monitoring, NE can be altered with imaging agents like magnetic nanoparticles or fluorescent dyes, enabling them as good theragnostic agents [106]. NE administered through different routes have demonstrated varying efficacy [101]. NE based oral formulation protects the medications from pH shifts, enzymatic breakdown, and dietary interactions improving its solubility, bioavailability, and absorption in the gastrointestinal tract [107]. For topical applications, NE enhance the skin’s ability to absorb hydrophobic substances by disrupting skin barriers and promoting penetration to the epidermis or dermis layer [108]. It has been used widely in skin cancer, skincare products, sunscreens, and for dermatological or cosmetic purposes [109, 110]. Intravenous NE serve as containers for chemotherapeutics, minimizing adverse effects, improving the effectiveness of treatment, and using both passive and active targeting to overcome multidrug resistance. In ophthalmic applications, NE enhance drug solubility and ocular bioavailability, improving efficacy and reducing dosing frequency in treating eye conditions [111, 112]. Pulmonary NE, delivered via inhalers or nebulizers, target the lungs for respiratory diseases like asthma or chronic obstructive pulmonary disease (COPD), enhancing mucosal interaction and drug bioavailability [101, 113]. Lastly, intranasal NE improve the delivery of antiviral agents against viruses such as dengue, respiratory syncytial virus (RSV), herpes, and human immunodeficiency virus (HIV), offering promising therapeutic potential in antiviral applications [101, 114].

### Mucoadhesion potential

**Fig. 3:**
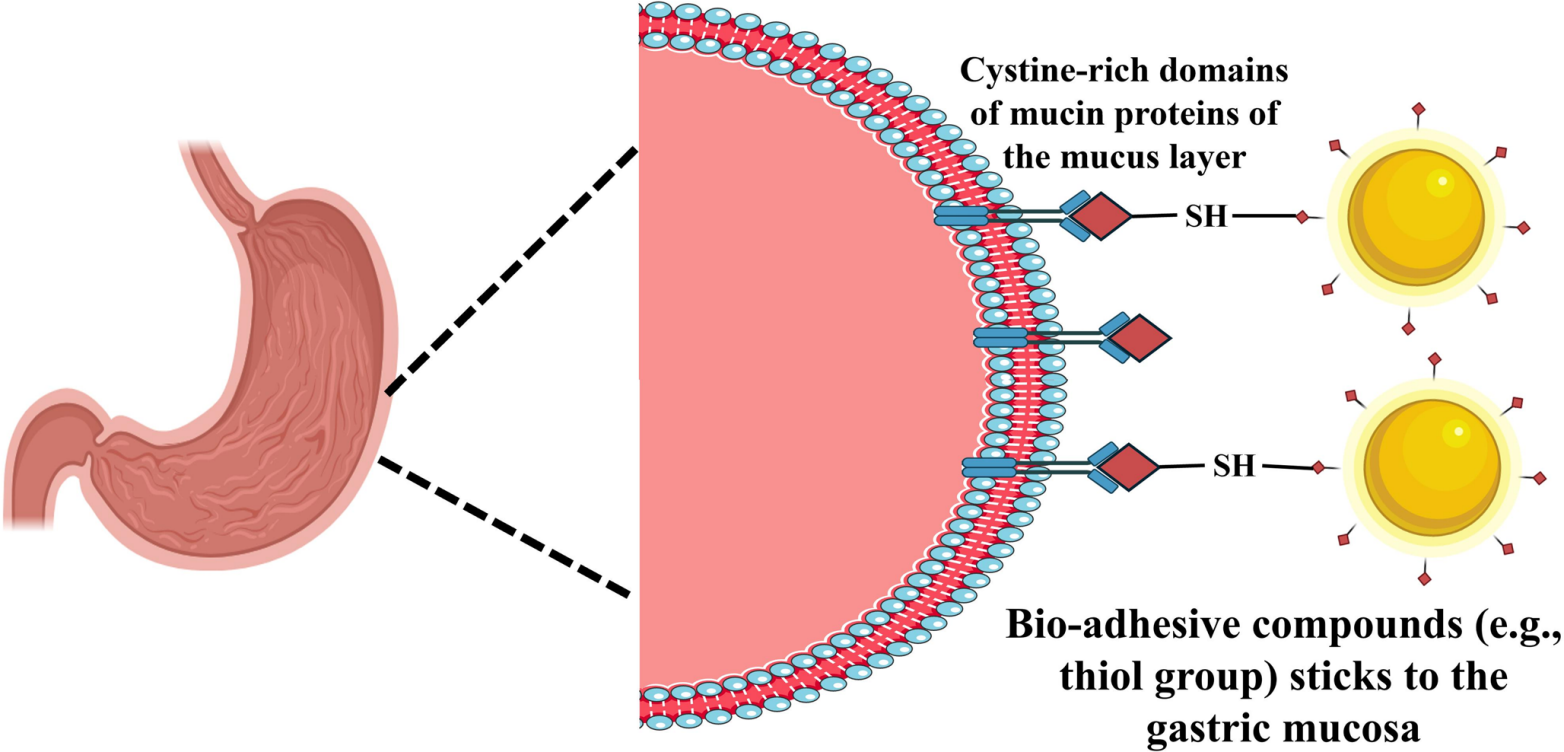
Permeation enhancement strategies to improve efficacy of nanoemulsion formulation in *H. pylori* infection

The mucoadhesion property of NE could be used to enhance the efficacy of *H. pylori* treatment. NE can promote mucus adhesion if a positively charged polymer like chitosan is used in the formulation. The negatively charged gastrointestinal mucus membrane will interact with the polymer and will increase the drug concentration available in the target site [115]. Lin et al., developed water in oil (W/O) NE loaded with amoxicillin for *H. pylori* eradication. Though amoxicillin is not a hydrophobic drug, it was found that NE was a better choice than other nanocarriers. Other nanocarriers failed to encapsulate low molecular weight hydrophilic drugs leading to rapid leaking and ultimately resulting in poor drug encapsulation. Studies have shown that chitosan heparin NE has facilitated mucoadhesion and release of encapsulated amoxicillin in the site of bacterial infection. Thus showing better bacterial clearance than the free drug solution that can be visualized by confocal and fluorescence imaging of the AGS gastric cancer cell line [116]. NE also has the ability to penetrate the mucus layer more effectively than conventional drug formulations due to its small size. This enhanced penetration allows NE-loaded drugs to reach the site of *H. pylori* infection more efficiently, improving their ability to eradicate the bacteria [117]. As shown in Fig. 3, carriers with a thiol functional group can create disulfide links with the mucosa, extending the duration of antibiotic payload exposure in the small intestine and increasing the likelihood of mucosal penetration [118]. Kanwal et al., developed a positively charged amphiphile containing NE for treating *H. pylori.* The positively charged amphiphilic surfactant containing NE of metronidazole prolonged the contact duration of the loaded drug to the *H. pylori* outer membrane increasing the transmembrane penetration of metronidazole ensuring better eradication of the bacteria [119]. Abdelhamid et al., reported that eugenol and cinnamaldehyde NE can easily penetrate through the cell wall of the bacteria and effectively enhance the anti*-H. pylori* activity [120]. Similarly, galangal NE formulated by Prasetya et al., was used for increased mucus penetration of the essential oil which further inhibited *H. pylori* growth successfully.

### Targeted delivery

**Fig. 4:**
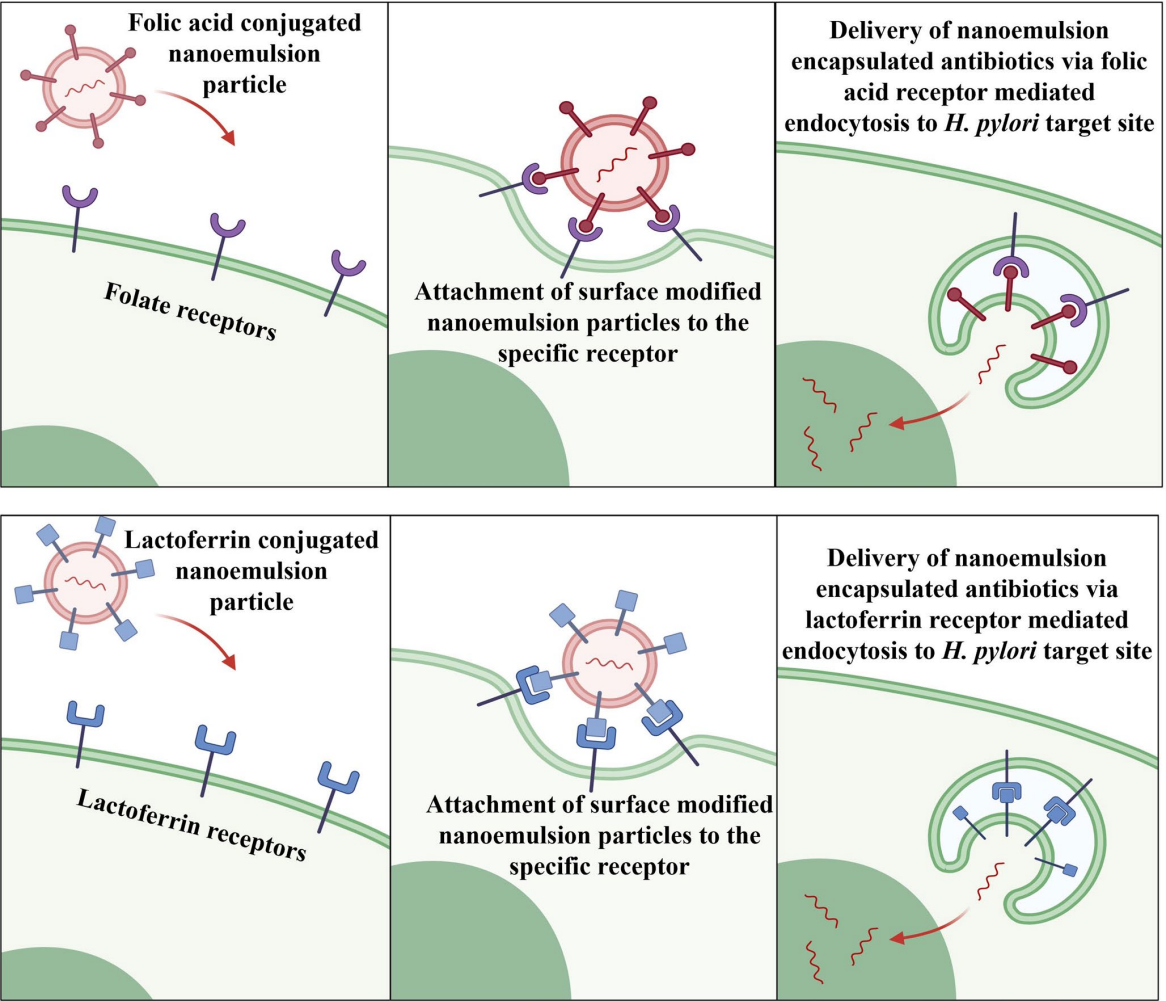
Surface modified nanoemulsion for targeted delivery to the bacterial site via receptor mediated endocytosis

NE can also act as carriers for targeting antibiotics to *H. pylori* bacteria by incorporating *H. pylori* specific ligands like Concanavalin A (lectin), folate and lactoferrin to the NE. This targeted delivery approach enables the NE-loaded antibiotics or antimicrobial agents to selectively accumulate at the site of infection, thereby enhancing their concentration locally and reducing off-target side effects [121]. Yang et al., fabricated an intranasal NE encapsulating an epitope peptide that delivers the drug directly to the infection site of *H. pylori* with the help of antigen-presenting cells (APCs) [122]. Conjugation on the NE surface can be done for selective attachment of the NE formulation to the bacterial surface receptors such as folate receptors. Folate receptors are overexpressed on the surface of *H. pylori*. By conjugating folate ligands to the NE droplets, site-specific targeting can be done. Further, the conjugated NE can be taken into the bacterial cells via receptor-mediated endocytosis. Thus, a drug can be directly delivered to the bacterial cells reducing the risk of resistance formation [123]. Also, folate-conjugated NE can be designed to release their payload in response to changes in pH enhancing their therapeutic effect [124]. Lactoferrin, a glycoprotein, is another ligand known to bind selectively to lactoferrin receptors on *H. pylori* bacteria. Thus, conjugating lactoferrin to the NE surface can enhance the targeted delivery of antibiotics to the bacteria. Lactoferrin can be conjugated on NE surface using covalent bonding and it will adhere to the bacterial surface through receptor-ligand interaction ensuring direct delivery of the drug to the target site [125–127]. Concanavalin A can be another targeting agent that specifically binds to the mannose residues present in the outer cell membrane of *H. pylori*. This selective binding facilitated localized drug delivery, leading to enhanced antibacterial effectiveness and more efficient bacterial eradication thus resulting in better antibacterial efficacy and efficient eradication [128]. Figure 4 represents how the surface modified NE can deliver the loaded drug to the target site of *H. pylori*.

### Controlled release

NE can also help in the controlled release of antibiotics over an extended period ensuring sufficient antibiotic concentration at the site. This prolonged release approach can lower the frequency of doses and increase patient compliance by maintaining therapeutic concentration of antibiotics in the bloodstream for a long time. Earlier studies showed that NE formulation allowed controlled release of clarithromycin, prolonging its presence in the gastric mucosa and optimizing the drug’s pharmacokinetic profile. This prolonged exposure of antibiotics enhanced its bactericidal activity against *H. pylori*, enhancing therapeutic results and lowered the possibility of antibiotic resistance [127]. This controlled release can be achieved by approaches like encapsulation of drug with oil droplets that will diffuse the drug in a controlled manner, incorporation of lipid matrices that dissolves gradually or by coating the NE with a polymer that degrades slowly, releasing the antibiotic in a controlled manner [129–132]. Saraf et al., developed hesperidin and clarithromycin nanostructured lipid carriers having a slow release rate even after 24 h and were shown to have two fold increase the rate of *H. pylori* eradication [133]. Herbal NE are also suitable for long-term use due to their gentle nature and minimal risk of dependence or tolerance buildup, allowing for extended benefits over time [134, 135].

### P-gp inhibition

**Fig. 5:**
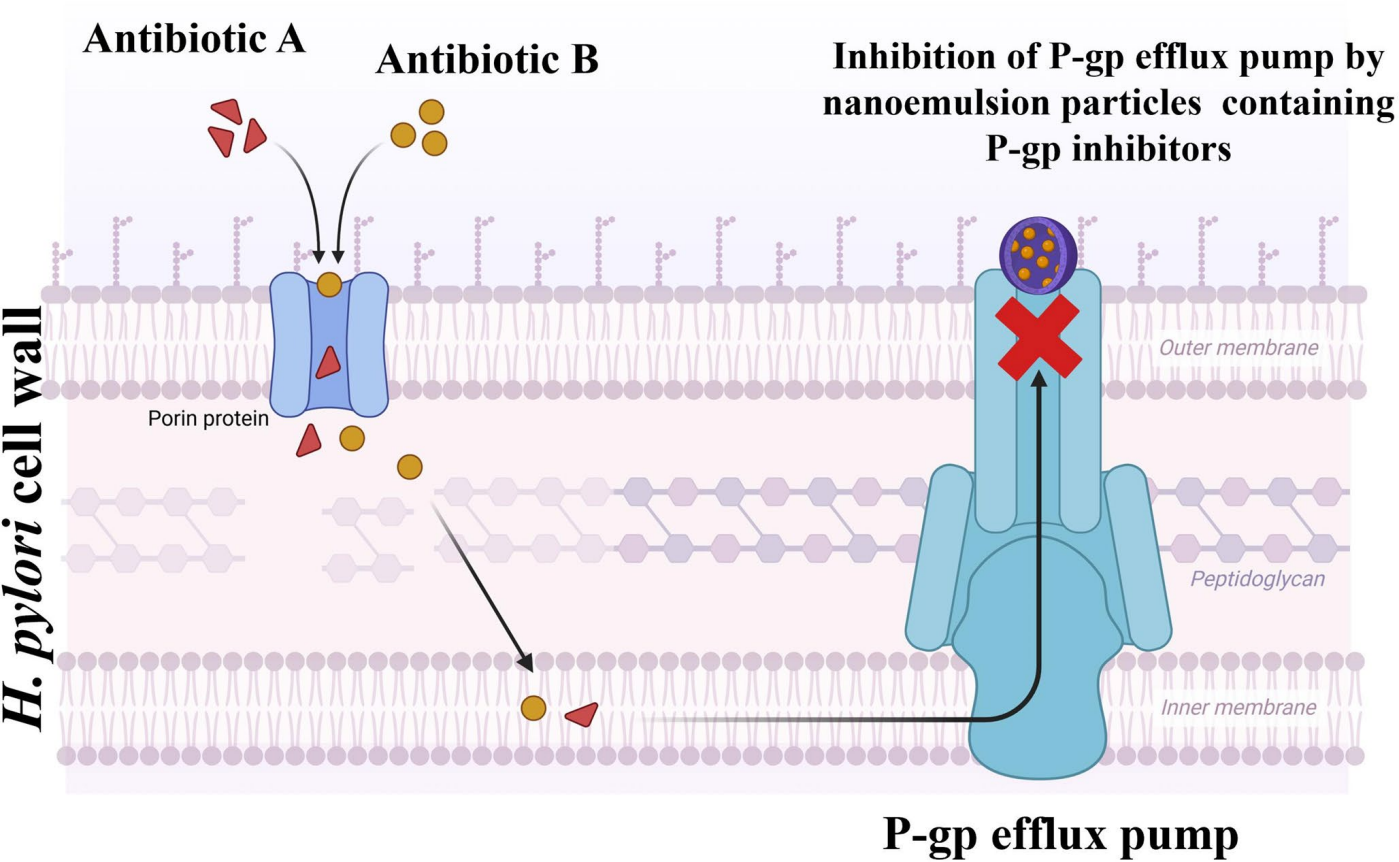
Inhibition of P-glycoprotein (P-gp) efflux pump by nanoemulsion

Macrolide antibiotics like clarithromycin, erythromycin, and azithromycin undergo P-glycoprotein (P-gp) efflux resulting in poor bioavailability. NE can play a significant role in inhibiting the P-gp efflux pumps. The P-gp efflux pump is a significant barrier in treating bacterial infections caused by *H. pylori*. These pumps, remove antibiotics out of bacterial cells, reducing the efficacy of the treatment [136]. Some components of NE can directly inhibit the activity of the P-gp efflux pump. For example, certain surfactants and lipids like cremophor EL, cetyltrimethyl ammonium bromide, tween-20, tween-80, and D-α-TPGS (tocopherol polyethylene glycol succinate 2000) can be used in the preparation of NE to inhibit the P-gp efflux pump [115, 137]. Other than that, NE can be incorporated with inhibitors of the P-gp efflux pump, such as verapamil, which competitively binds to the P-gp efflux pump, reducing its ability to efflux out antibiotics [138, 139]. Figure 5 illustrates the mechanism of inhibition of the P-gp efflux pump by NE, demonstrating its impact on enhancing drug accumulation within cells.

### Co-delivery

NE offers a platform for combining multiple antimicrobial agents or therapeutic agents with complementary mechanisms of action in a single formulation. By encapsulating different drugs within the same NE formulation, synergistic effects can be achieved, which is important to improve *H. pylori* eradication and reduce the risk of antibiotic resistance. For example, the combination of erythromycin and metronidazole in a NE-based delivery system has shown enhanced antimicrobial activity against *H. pylori* [85]. NE can combine antibiotics with adjuvant therapies like the inclusion of antioxidants or anti-inflammatory agents that can provide dual therapeutic benefits. NE can also incorporate natural compounds known for their gastroprotective properties, such as essential oils or plant extracts. These natural agents can work synergistically with the antibiotic to protect the stomach lining, promoting the healing of existing gastric ulcers and protect against the formation of new ulcers while combating *H. pylori*. For example, Mosallam et al., fabricated a combination delivery of curcumin and clarithromycin NE, which showed better biofilm inhibition and synergistic effect and improved bacterial clearance [85]. Urease inhibitors have been reported as a good adjuvant with antibiotics for the effective eradication of *H. pylori* [140, 141]. Co-delivery of a potent urease inhibitor with antibiotics can be achieved with the NE formulation.

### Gastroprotective effect

**Fig. 6:**
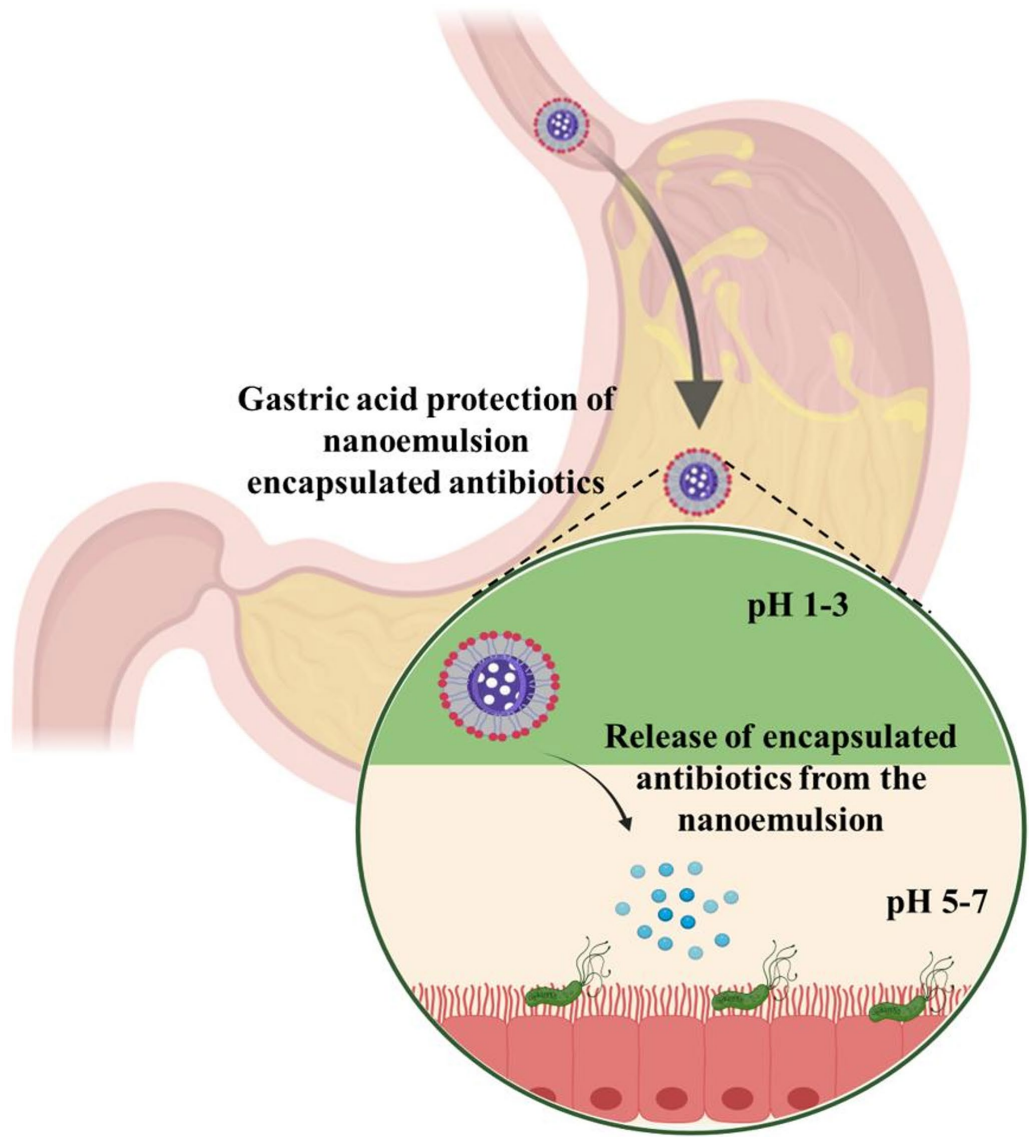
Gastroprotective effects of nanoemulsion for *H. pylori *eradication

NE offers gastroprotective benefits by shielding encapsulated drugs like clarithromycin and amoxicillin from the acidic stomach environment, reducing gastric irritation and maintaining drug stability until it reaches the target site (Fig. 6). This enhances the therapeutic efficacy against *H. pylori* [142]. For example, Saraf et al., developed a NE formulation with hesperidin and clarithromycin to improve solubility and protect the drugs from stomach acid, achieving better bacterial eradication [133]. Apart from that, NE can also minimize gastric irritation and inflammation by incorporating anti-inflammatory agents or using lipids or surfactants to reduce pro-inflammatory cytokines. Researchers have formulated curcumin-clarithromycin NE system that demonstrated enhanced *H. pylori* eradication compared to free drugs, showcasing the advantages of encapsulating antibiotics in NE for gastric protection [143].

### Bioavailability enhancement

**Fig. 7:**
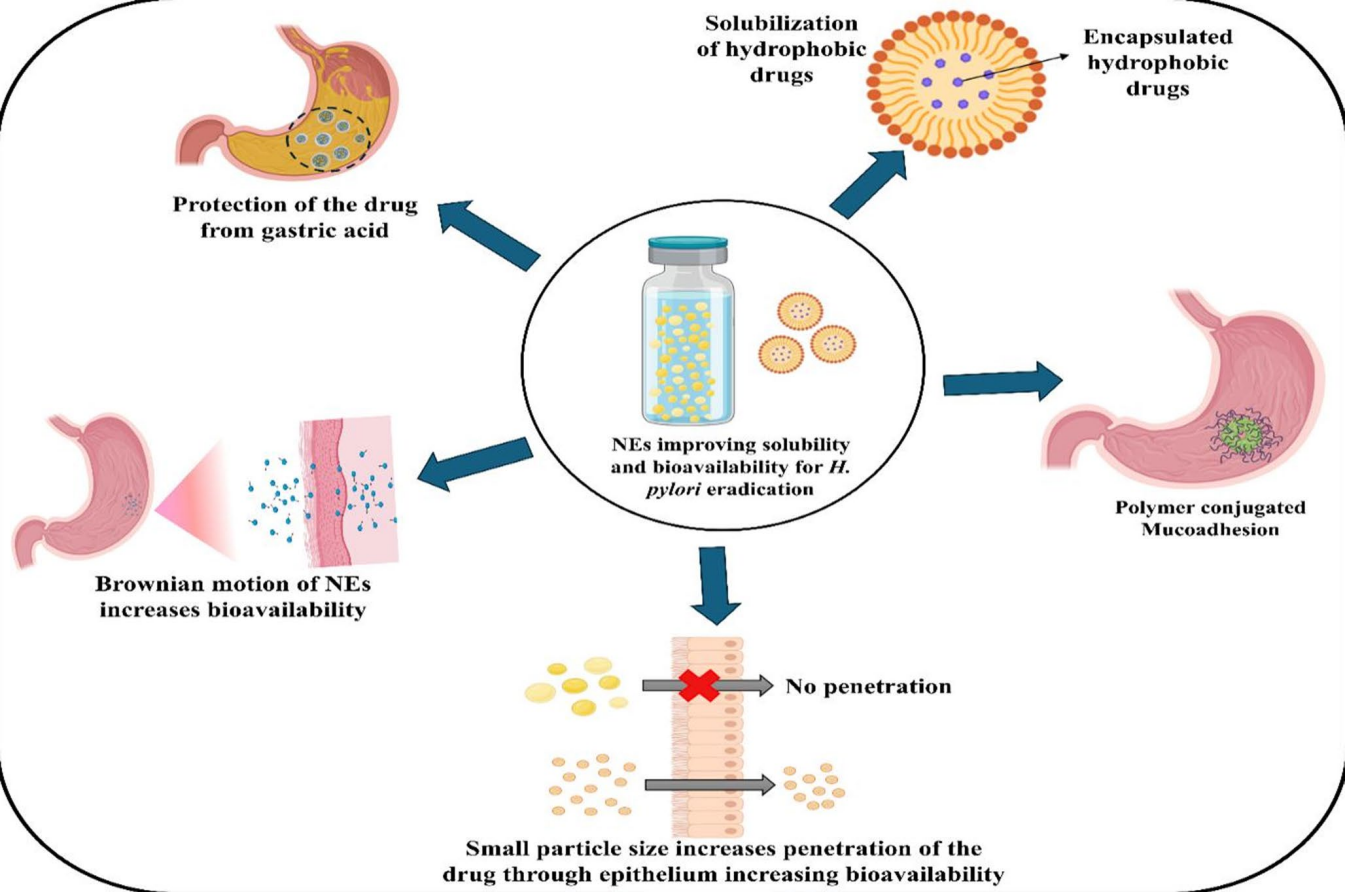
Potential mechanisms by which nanoemulsion can improve solubility and bioavailability of antibiotics for effective eradication of *H. pylori*

The potential of NE to enhance the solubility could be utilized to improve the bioavailability of poorly water-soluble antibiotics like clarithromycin, erythromycin, levofloxacin, and rifabutin which are widely used in the *H. pylori* treatment. Hydrophobicity limits these drugs from reaching the target site. NE can offer enhanced solubility, and bioavailability of lipophilic drugs, and can be considered as an effective drug carrier [115]. Figure 7 depicts various potential mechanisms offered by NE for overcoming the solubility and bioavailability challenges to improve treatment efficacy. NE increase the solubility of hydrophobic drugs by encapsulating them in oil phase, which is dispersed in an aqueous medium [144]. Certain surfactants also have the ability to increase the contact angle between the drug and aqueous phase, promoting better drug distribution. This creates a large surface area that allows the drug to be more readily dissolved. This increased surface area and small particle size leads to faster and more efficient absorption of the drug into the bloodstream. Small droplets of NE facilitate easier absorption, better distribution, and movement in the body due to Brownian motion resulting in higher bioavailability [145]. Tran et al., prepared a NE-based delivery system for erythromycin, a drug that is prone to acidic degradation and has a limited solubility in water for treating *H. pylori*. The results showed a 4-fold increase in efficacy of the encapsulated drug in comparison to free erythromycin. By formulating clarithromycin into a NE, its solubility was significantly improved due to the small droplet size and increased surface area provided by the NE structure. Thus, when administered orally, the NE containing clarithromycin can rapidly disperse in the gastrointestinal tract, allowing enhanced absorption of the drug compared to conventional formulations leading to increased therapeutic efficacy [133]. Mosallam et al., developed an oil in water (O/W) NE co-delivering curcumin and clarithromycin to combat the conventional limitations of *H. pylori* treatment. The NE enhanced the bioavailability of both the lipophilic drugs against the bacteria [143]. Another study by Hidalgo et al., reported that NE of curcumin showed better inhibitory effect against the *H. pylori* in comparison to the free drug, and also effectively inhibited bacterial biofilm formation [146]. By optimizing drug-loading capacity and pharmacokinetic profiles, NE represents a promising strategy to overcome bioavailability barriers in *H. pylori* therapy, potentially reducing treatment duration and improving patient compliance. Their adaptability across oral, topical, and intravenous routes further underscores their versatility in advancing antimicrobial formulations.

### Safety and toxicity

Reports show that NE are increasingly used in oral drug delivery due to their ability to improve solubility, absorption and bioavailability. NE have been reported to improve efficacy against *H. pylori* by enhancing mucosal penetration and drug stability [147]. However, there are also reports showing that the nanometric size and the formulation excipients like surfactants leads to safety and toxicity concerns. Surfactants may irritate the intestinal mucosa causing irritation and diarrhoea. Components of co-surfactants like ethanol, polyethylene glycol (PEG) and solubilizing agents may cause local toxicity at high doses. It is also possible that small particle size may facilitate uptake to cells or tissues like liver and spleen leading to tissue toxicity [148, 149]. There are also reports showing that NE may also disrupt the gut microbiome [150, 151]. This will be an added challenge, as the *H. pylori* infection itself will lead to dysbiosis, inflammation and compromised gastric barriers [152]. While targeting *H. pylori* is desired, further disruption of an already compromised microbiota may worsen gastric inflammation or delay mucosal healing [153, 154]. These safety concerns pose challenge in the applicability of NE for oral use and needs to be addressed effectively. This will include careful selection of excipients, particle size optimization, in-vitro and in-vivo toxicity studies and microbiome compatibility testing [155]. While selecting the excipients, it is recommended to use only generally recognized as safe (GRAS) listed excipients and prefer natural lipids like olive oil and mild surfactants like lecithin and polysorbates. It is recommended to avoid and minimize the use of ethanol and synthetic polymers [148]. In order to avoid the cellular uptake by endocytosis and to reduce systemic translocation, it is recommended to avoid particle size less than 100 nm. The problem of gut microbiome disruption can be addressed by designing targeted NE that release the drug specifically at the site of infection, minimizing exposure to intestinal microbiota [156]. The use of mucoadhesive systems can help to retain the NE longer in stomach and can avoid unwanted effects in the intestine [157]. Co-administration of probiotics along with antibiotics loaded NE is also a strategy to restore and maintain gut microbial balance during and after therapy [158]. Use of biodegradable surfactants or lipid excipients in NE can enable sustained and localised drug release in the stomach preventing the intestinal spread [159]. It is also advisable to assess microbiota composition pre and post treatment using 16 S rRNA sequencing. Finally, it is advisable to conduct in-vitro and in-vivo toxicity studies [160]. Cytotoxicity studies using intestinal epithelial cell lines (Caco-2, HT-29) and acute and subacute oral toxicity studies in the animal models should be conducted to monitor signs of GI irritation, changes in liver/kidney markers, immune markers and histopathological changes [161].

The above discussions clearly highlight the advantages, usefulness and safety concerns of NE formulation in treating the *H. pylori* infection. NE provides these advantages because of its diverse capabilities such as enhanced drug solubility, improved bioavailability, biocompatibility, controlled release, and targeted delivery. These properties ensure that therapeutic agents are effectively transported to the site of infection, thereby maximizing their efficacy. Antibiotics used in the first- and second-line therapies could be formulated as NE to enhance the treatment efficacy and eradication of *H. pylori* infection. Considering these advantages, many earlier workers have attempted to prepare NE and evaluated them for their potential for treating *H. pylori* infection. In Table 4 we summarise the studies detailing the methodologies employed, limitations, reproducibility, models used and the efficacy data. All these studies were either in-vitro or pre-clinical evaluations. As on date no studies have reported the use of NE in clinical settings.

**Table 4:** Summary of reported studies on the preparation and evaluation of nanoemulsion for *H. pylori* treatment

Drug encapsulated	Type of NE	Method of preparation	Reproducibility	Model used (in-vitro cell lines/in-vivo)	Route of drug delivery	Mechanism of action	Comparison of pharmacokinetics with free drug	Comparison of efficacy with the free drug	Limitations	Clinical trial phase	References
Curcumin	O/W	Oil phase (medium-chain triglyceride oil with curcumin) was mixed with aqueous phase containing surfactant (Tween 80) and co-surfactant (propylene glycol), followed by probe sonication at 40% amplitude for 5 min with intermittent cooling	High	in-vitro: • GES-1 (human gastric epithelial cells) for cytotoxicity, adhesion, and internalization assays • *H. pylori* reference strains for antibacterial activity and biofilm inhibition	Oral	• Inhibits *H. pylori* adhesion and internalization into gastric epithelial cells; disrupts biofilm formation by *H. pylori*. • Reduces IL-8 secretion in infected GES-1 cells, indicating suppression of *H. pylori* -induced inflammation • Scavenges reactive oxygen species (ROS) induced by infection	• Improved stability of curcumin in simulated gastric fluid and protection from degradation compared with free curcumin • Enhanced solubility and cellular uptake in vitro. • No direct in-vivo pharmacokinetics (absorption, plasma levels, etc.) measured in this work; focus was on in vitro stability, permeability, and activity	• Nanoemulsified curcumin was significantly more effective than free curcumin • Lower MIC (minimum inhibitory concentration) against *H. pylori* • More potent at inhibiting biofilm formation • Greater suppression of IL-8 production (anti-inflammatory) in gastric epithelial cells	• No in-vivo animal model: All biological assays were in vitro using gastric cell lines and bacterial cultures. • No clinical data: Results are not yet confirmed in animal infection models or human studies. • Potential scale-up and stability in real gastric environment need further assessment	Not applicable	[146]
Curcumin and Clarithromycin	O/W	• Oil phase: Curcumin (100 µg/mL) dissolved in coconut oil (15% v/v) with Tween 80 (10% v/v, surfactant) and propylene glycol (10% v/v, co-surfactant). • Aqueous phase: Clarithromycin (100 µg/mL, dissolved in 10% DMSO and 90% water). • The aqueous phase added dropwise to oily phase using a mixing homogenizer to form a pre-emulsion. • Pre-emulsion homogenized at 10,000 rpm for 30 min at 25 °C. • Followed by high-energy ultrasonication for 30 min to finalize NE.	High reproducibility	in-vitro: • Human gastric epithelial cells (various *H. pylori* strains including clinical isolates and ATCC standard strain) for MIC, antibiofilm, and antibacterial assays. in-vivo: • CD-1 male mice infected with *H. pylori* for efficacy and histopathological assessments.	Oral	• NE protects curcumin and clarithromycin from acidic stomach environment, improving stability and bioavailability. • Curcumin disrupts bacterial membranes, inhibits adhesion and biofilm formation, and reduces inflammation (suppresses IL-8 secretion). • Clarithromycin acts as antibiotic targeting bacterial protein synthesis. • Synergistic antibacterial effect observed with co-delivery. • Controlled release in intestinal pH facilitates targeted drug release. • Histological improvement observed in gastric tissues with reduced inflammation, necrosis, and mucosal damage	• Cur-CLR-NE shows improved stability in gastric juice with over 90% drug retained under acidic conditions, compared to rapid degradation of free drugs. • At intestinal pH (~ 7.2), controlled drug release observed within 15 min. • Enhanced solubility and cellular uptake in-vitro. • No direct systemic pharmacokinetic (absorption/plasma) data reported in this stud	• Cur-CLR-NE exhibited significantly lower minimum inhibitory concentrations (MICs 6.25 to 12.5 µg/mL) than free curcumin or clarithromycin alone. • Greater inhibition of biofilm formation at sub-MIC levels. • In-vivo, NE achieved higher *H. pylori* clearance than free drugs at the same dosage and regimen. • Superior reduction in gastric inflammation and tissue repair compared to free drug groups	• Lack of systemic pharmacokinetic data and human clinical trials. • In-vivo study limited to mouse model with short treatment duration. • Further long-term safety, toxicity, and clinical efficacy assessments needed.	Not applicable	[143]
Amoxicillin	W/O	High-pressure homogenization using an homogenizer (10,000–15,000 rpm), followed by high-pressure homogenization for droplet size reduction	High	In-vitro: • GES-1 gastric epithelial cells for uptake and cytotoxicity In-vivo: • BALB/c mice infected with *H. pylori*	Oral	• Prolonged gastric residence time via mucoadhesive chitosan coating • Enhanced drug delivery to infection site via sustained release • Inhibition of bacterial adhesion to gastric cells	• Prolonged drug release, higher gastric retention, and significantly • Greater bioavailability at the site of infection compared to free amoxicillin	• Higher *H. pylori* clearance rate in mice • Lower bacterial counts in stomach tissue • Better therapeutic index and reduced systemic exposure	• No long-term toxicity study • Limited data on human translation • pH sensitivity and W/O stability under digestive stress not deeply explored	Not applicable	[116]
Erythromycin	O/W	Oil and aqueous phases were separately heated and mixed under high shear then passed through a high-pressure homogenizer at elevated temperature (above erythromycin’s melting point)	Good	In-vitro: • AGS (gastric epithelial) cell lines Ex-vivo: • Mouse gastric tissue homogenate for evaluating stability and activity	Oral	• Enhanced epithelial penetration and retention • Synergistic activity from surfactant–drug interaction • Nanoencapsulation protected erythromycin from degradation in acidic gastric conditions	While full PK profiling wasn’t done, erythromycin-loaded NE showed significantly better acid stability, suggesting improved gastric retention and sustained local exposure	• Minimum inhibitory concentration (MIC) of NE formulation was 4× lower than free erythromycin • Strong synergistic effect observed when co-administered with other bioactives (e.g., acid suppressants)	• In-vivo PK or toxicity studies • No long-term stability testing under physiological conditions • Human applicability not validated	Not applicable	[162]
Eugenol and Cinnamaldehyde	O/W	• Essential oils (Eugenol/Cinnamaldehyde or their mixture) were used as oil phase • Surfactants (Tween 80) and ethanol were included in aqueous phase • Aqueous phase added to oil phase under stirring	Moderate	In-vitro only: • *H. pylori* inoculated into yogurt • Antimicrobial activity of NE and probiotics tested in contaminated yogurt matrix	Oral	• Disruption of *H. pylori* membrane integrity • Eugenol and cinnamaldehyde act via lipid peroxidation, efflux pump inhibition, and biofilm disruption • Combined with probiotics for synergistic inhibition	No pharmacokinetic study conducted	• Combined NE of eugenol + cinnamaldehyde reduced *H. pylori* count by 3.9 log CFU/g • Greater reduction compared to NE or probiotic alone • Maintained acceptable counts of starter culture and probiotics in yogurt	• No in-vivo or human studies • No toxicity or sensory evaluation for NE at higher doses • Study limited to food model system (yogurt) only	Not applicable	[120]
*Cinnamomum zeylanicum* (Cinnamon) Essential Oil	O/W	• Low-energy emulsification using variable surfactant concentration • Droplet sizes tuned using different surfactant-to-oil ratios	Low	Ex-vivo: • Clinical isolates from gastric biopsies (5 patients, Shariati Hospital, Tehran	Not directly applied in-vivo. Ex-vivo model suggests oral or gastric route potential	• Rapid membrane disruption of *H. pylori* • Quantified by protein and nucleic acid release (OD280 and OD260) • SEM showed membrane collapse and lysis • Stronger efficacy with smaller droplet size	Not conducted – PK profile not assessed	• NE showed comparable efficacy to 70% ethanol • Achieved complete bacterial inhibition at 100 µg/mL in 2.5 min • Stronger and faster than larger droplets (F2) or untreated controls	• No in-vivo testing or toxicity profile • Long-term stability and interaction with gastric mucosa not explored • Not directly compared with standard antibiotics like clarithromycin	Not applicable	[163]
Azithromycin and Curcumin	O/W	High-shear emulsification or spontaneous emulsification formulated with natural oils such as clove oil	Moderate	In-vitro: • MIC, FICI, biofilm assays, gene expression (babA, hopQ, ureA) In-vivo: • Mouse challenge model with histopathology of gastric tissue	Oral	• Synergistic antimicrobial action via membrane interaction, biofilm inhibition, and virulence gene suppression • Downregulation of key virulence genes (e.g., babA, hopQ, ureA) • Molecular docking showed strong interaction of curcumin with *H. pylori* target proteins	Not directly measured; however, NE allowed sub-MIC dosing of curcumin to enhance azithromycin’s effect, suggesting improved local delivery	• Curcumin NE and azithromycin showed synergistic effects • Superior biofilm inhibition and tissue protection vs. either agent alone	• No human studies • PK not established • Safety of long-term curcumin NE co-administration remains to be evaluated	Not applicable	[164]
HpaA epitope peptide	O/W	• Epitope peptide incorporated into aqueous phase • Oil and surfactant phase prepared separately • Mixtures homogenized and stabilized to form NE	Moderate	In-vivo: • *H. pylori*-infected BALB/c mice **Immunological assays**: • Th1 response, cytokine profiling, colonization burden	Intranasal	• Enhanced mucosal antigen delivery and prolonged nasal residence time • Boosted Th1-biased immune response • Improved cellular uptake of antigen • Significant reduction in gastric *H. pylori* colonization even without CpG adjuvant	Not directly assessed; however, NE showed extended antigen release and enhanced mucosal uptake	• NE-P22 alone significantly reduced bacterial load • Efficacy further increased when combined with CpG • Outperformed peptide alone or control NE without peptide	• No human studies • No systemic toxicity or long-term mucosal safety assessment • Translation to human mucosal immune system remains uncertain	Not applicable	[165]
Tea Tree Oil	O/W	• Tea tree oil, surfactant, co-surfactant mixed and diluted • Chitosan added for surface functionalization and stability	Limited	In-vitro: • *H. pylori*, MRSA, and P. aeruginosa In-vivo: • Mouse models with gastric ulcers (for *H. pylori*)	Oral	• Antibacterial synergy: Disruption of bacterial membrane • Metabolomic disruption: Altered glyoxylate, glutamate, tryptophan metabolism • Downregulation of 274 bacterial proteins; upregulation of 251 others	Not explicitly measured; however, NE showed superior stability, tissue penetration, and enhanced delivery over aqueous suspensions	• NE exhibited 2–8× higher antibacterial efficacy than equivalent tea tree oil suspension • Significant in-vivo bacterial clearance in both infection models	• No long-term toxicity or human study • No direct PK/PD profile comparison • Specific mechanisms of chitosan-bacteria interaction remain unclear	Not applicable	[166]
Ethanolic extract from *Casearia sylvestris* leaves	O/W	Prepared using a standard emulsion-evaporation method and incorporated with the ethanolic extract	Good	In-vitro: • *H. pylori* ATCC 43,504 (MIC, biofilm inhibition, time-kill assays) In-vivo: • Male Wistar rats experimentally infected with *H. pylori*	Oral	• Disruption of *H. pylori* viability and biofilm formation • Ethanolic extract was most effective in bacterial eradication • Likely action through terpenoid-induced membrane disruption and anti-inflammatory properties	Not assessed	Increased anti-biofilm activity, and bacteriostatic effect in-vivo studies. It eliminated the pathogen from the gastric mucosa and reduction of the ulcerative lesion was observed.	• NE blunted direct in-vitro activity in microdilution assay • No PK or toxicological analysis • Short-term evaluation in animal mode	Not applicable	[167]
Hesperidin (Hesp) and Clarithromycin (CLR)	O/W	• Hot homogenization followed by ultrasonication • Combination of solid lipids (Compritol 888 ATO), liquid lipids (Capryol), and surfactants (Tween 80) • Drugs were dissolved in lipid phase and dispersed in aqueous phase	High	In-vitro only: • *H. pylori* culture for MIC testing • Imaging flow cytometry for interaction studies	Oral	• Adherence of NLC to *H. pylori* membrane • Disruption of membrane integrity leads to cytoplasmic leakage • Sustained release increases exposure time and drug accumulation at bacterial sit	Not measured; however, sustained and controlled release was confirmed in-vitro	• Enhanced antibacterial activity compared to single drugs • Synergistic effect observed when hesperidin and clarithromycin were combined	• No in-vivo or clinical testing • Short-term assessment only • Cytotoxicity and immune response not evaluated	Not applicable	[133]
Dextran sulphate and Curcumin	O/W	• Oil-core NE were prepared using a spontaneous emulsification technique • Co-stabilized with lecithin and lysozyme • Coated with low (DexS40) or high (DexS500) MW dextran sulfate, or chitosan for charge tuning	High	In-vitro: • *H. pylori* adhesion assay using AGS (gastric adenocarcinoma) cells • Cytotoxicity tests using AGS, Caco-2, and MDCK cell lines	Not tested in-vivo, but designed for oral gastric delivery	• Inhibition of *H. pylori* adhesion to gastric epithelial cells • Electrostatic repulsion by negatively charged DexS40-NC • Curcumin may add anti-inflammatory effects, but adhesion inhibition is the primary action	Not conducted	• DexS40-NC significantly inhibited adhesion of *H. pylori* to AGS cells • Performed better than DexS500-NC, plain NE, or chitosan-coated NE • Curcumin-free NE showed reduced efficacy, supporting role of encapsulated phytochemical	• No in-vivo validation • Not tested in infection or eradication models • Effectiveness in mucosal environments and digestive stability not evaluated	Not applicable	[168]
*Alpinia galanga* (Lengkuas) essential oil	O/W	Magnetic stirring followed by ultrasonication	Moderate	In-vitro only: • *H. pylori* bacterial inhibition via well diffusion assay	Not tested in-vivo, but oral application implied	antibacterial effect likely due to essential oil disrupting bacterial membranes and inhibiting growth	Not assessed	• Inhibition zone of 9.5 mm at 1% NE concentration • Compared to control and non-NE oil, NE showed improved dispersion and efficacy	• No in-vivo or animal model testing • No toxicity or stability evaluation • Lack of pharmacokinetic/pharmacodynamic data	Not applicable	[169]
Clarithromycin	O/W	• Prepared using High speed homogenization • Polysorbate 80 (surfactant), olive oil (oil phase), polyvinyl alcohol (PVA, co-surfactant	High	In-vitro only • Antibacterial activity against *H. pylori* strains via well-diffusion assay	Oral	• Enhanced solubility and dispersion of poorly water-soluble clarithromycin • Facilitated increased drug–bacteria contact and biofilm penetration	Not assessed	• NESH 01 showed larger inhibition zones than free clarithromycin • Indicates increased antimicrobial potency from NE formulation	• No in-vivo or animal testing • No cytotoxicity/safety profiling • No pharmacokinetic assessment	Not applicable	[170]
Metronidazole	O/W	• Positively charged amphiphile (alkylated morpholine) added for enhanced mucosal adhesion • Emulsification optimized through solubility, particle size, and zeta potential evaluations	High	In-vitro: • *H. pylori* culture • Potential for future in-vivo application noted but not evaluated in this study	Oral	• Positive surface charge enhanced interaction with negatively charged gastric mucosa • Increased local concentration of metronidazole near *H. pylori* colonization sites • Facilitated drug penetration and biofilm disruption	Not evaluated directly; however, improved solubility and dispersibility observed	Higher antibacterial activity than free metronidazole in vitro	• No in-vivo validation • Lack of data on mucosal safety, toxicity, or PK profile • No long-term stability assessment	Not applicable	[119]
Thymoquinone (TQ)	O/W	• Almond oil (oil), Tween 80 (surfactant), PEG 200 (co-surfactant) • Prepared by mixing oil/surfactant/cosurfactant with TQ, then dispersing in aqueous medium using magnetic stirrer	Moderate	In-vivo only: • Indomethacin-induced gastric ulcer model in Wistar rats	Oral	• Improved gastroprotective effect due to enhanced solubility and bioavailability of TQ • Antioxidant and cytoprotective activity against NSAID-induced damage	Not assessed	• 2-fold higher gastroprotective index vs. non-emulsified TQ • Reduced ulcer index and better mucosal healing	• No bacterial eradication model (e.g., *H. pylori*) tested • PK/PD parameters not quantitatively analysed • Only one animal model studied	Not applicable	[171]

## Conclusion

Effectively managing *H. pylori* infection remains a significant clinical challenge due to the organism’s ability to persist within the acidic environment of gastric mucosa, contributing to ineffective treatment. It is seen that the ineffective treatment leads to the development of resistance, PUD and gastric cancer. This review explicitly analysed the potential of nanoemulsion in combating the challenges related to effective eradication of the organism. Beyond their basic role as drug carriers, NE offers unique capabilities that can address key obstacles in *H. pylori* therapy. It is evident from this review that NE can help in increasing the bioavailability at the gastric mucosa leading to localized action at the infection site because of its mucoadhesive properties, tuneable droplet size, and compatibility with both hydrophilic and lipophilic agents that facilitates prolonged gastric residence. The capacity of NE to incorporate targeting ligands such as Concanavalin A, lactoferrin etc., can help in the targeted delivery of antibiotic to the organism. The ability of the NE to simultaneously deliver multiple drugs like an antibiotic and a non-antibiotic adjuvant (Urease inhibitors, anti-inflammatory agents, gastroprotective agents etc.) can open avenues for synergistic, multi-modal eradication strategies. The controlled and sustained drug release conferred by NE further enhances local drug concentration while minimizing systemic exposure and adverse effects, with additional benefits including gastroprotection and improved stability of sensitive drugs in the acidic gastric environment. NE also found to offer advantage of cost-effectiveness, making them a practical and smart drug delivery system for *H. pylori* treatment. Early in-vivo studies have demonstrated an enhanced bacterial clearance and histological recovery in infected animal models, signalling clinical promise. In conclusion, nanoemulsion as a drug delivery option for *H. pylori* treatment have the potential to effectively eradicate the organism and can emerge as an efficient and safe solution to the growing challenge of antibiotic resistance. Research in this direction should be carried out to develop nanoemulsion incorporating the antibiotics, targeting agents and other promising non antibiotic adjuvants for eradication of the infection.

## Future directions

Despite NE represent a highly promising platform for targeted drug delivery against *H. pylori*, it is important to note that majority of the current evidence on the usefulness of NE as a promising drug delivery option is derived from the in-vitro and preclinical studies in animal models. As on date there are no reports on the efficacy of NE as a drug delivery option against *H. pylori* in a clinical setting. The reasons for little or no use of NE in a clinical setting is attributed to the stringent regulatory requirements for taking a drug product to clinical trials. In case of NEs, precise characterization of droplet size, polydispersity index and surface charge are technically challenging. Small variations in these physicochemical parameters can drastically affect pharmacokinetics, safety and efficacy. As yet, no advanced pharmacokinetics studies demonstrating the in-vivo fate (ADME) of NE have been conducted. At the same time these physicochemical data is an important part of the regulatory submission seeking clinical trial permission. Future studies are needed to fully characterize NE and to evaluate the thermodynamic stability as per ICH protocols to ensure consistent drug delivery throughout the clinical trial period. It is also a prerequisite to evaluate the safety and toxicity of surfactants and co-solvents used in the preparation of NE to understand the long-term effects, tissue distribution and any possible immunogenic reactions. Scalability of manufacturing of NE without altering their properties is also a big challenge because minor change in process parameter can affect droplet size and stability leading to batch-to-batch variation. The lack of guidelines specific to NE is also a reason preventing the transition of NE to clinical trials. Thus, while NE show great promise for drug delivery, their successful bench-to-bedside translation hinges on overcoming substantial challenges related to stability, scalability, and regulatory compliance.

## Data Availability

Data sharing is not applicable to this article as no data set was generated or analysed during the current study.
